# Major bioactive phenolics in *Bergenia* species from the Indian Himalayan region: Method development, validation and quantitative estimation using UHPLC-QqQ_LIT_-MS/MS

**DOI:** 10.1371/journal.pone.0180950

**Published:** 2017-07-27

**Authors:** Renu Pandey, Brijesh Kumar, Baleshwar Meena, Mukesh Srivastava, Tripti Mishra, Vandana Tiwari, Mahesh Pal, Narayanan K. Nair, Dalip K. Upreti, Tikam Singh Rana

**Affiliations:** 1 Sophisticated Analytical Instrument Facility, CSIR-Central Drug Research Institute, Lucknow, India; 2 Academy of Scientific and Innovative Research (AcSIR), New Delhi, India; 3 Plant Diversity, Systematics & Herbarium Division, CSIR-National Botanical Research Institute, Lucknow, India; 4 Biometry and Statistics Division, CSIR-Central Drug Research Institute, Lucknow, India; 5 Phytochemistry Division, CSIR-National Botanical Research Institute, Lucknow, India; International Nutrition Inc, UNITED STATES

## Abstract

*Bergenia* species are important medicinal plants used in indigenous systems of medicine for their antilithiatic and diuretic properties. An ultra-high performance liquid chromatography coupled to hybrid linear ion trap triple quadrupole mass spectrometry (UHPLC-QqQ_LIT_-MS/MS) method has been developed and validated for the estimation of quantitative variation of eight major bioactive phenolics in the rhizomes (150 samples) of four species of this herb, *Bergenia* (*B*. *ciliata*, *B*. *ligulata*, *B*. *purpurascens* and *B*. *stracheyi*). Chromatographic separation was obtained on a Waters ACQUITY UPLC^TM^ BEH (ethylene bridged hybrid) C_18_ column with a mobile phase consisting of 0.1% (v/v) formic acid aqueous solution and acetonitrile under a gradient elution manner. A hybrid linear ion trap triple quadrupole mass spectrometer was operated in negative electrospray ionization mode with multiple reactions monitoring for detection and quantification of the eight compounds. The validated method demonstrated good linearity (r^2^ ≥ 0.9991), precision (RSD ≤ 1.87%) and accuracy (95.16–102.11%, RSD ≤ 1.83%) for all reference analytes. The quantitative results revealed that *B*. *ligulata* contains the highest amount of the major active marker-bergenin. The results also suggest that sensitive UHPLC-QqQ_LIT_-MS/MS method, a sensitive, accurate and convenient one, could be helpful in identification of potential accession(s), rapid quality control and establishing authenticity of *Bergenia* species as raw material for pharmaceutical industries.

## Introduction

The genus *Bergenia* Moench (Saxifragaceae) consists of about 10 species of perennial rhizomatous herbs distributed chiefly in the temperate and subtropical regions of Central and East Asia [1]. The four species of *Bergenia viz*., *B*. *ciliata* (Haw.) Sternb., *B*. *ligulata* (Wall.) Engl., *B*. *stracheyi* (Hook.f. & Thoms.) Engl., and *B*. *purpurascens* (Hook.f. & Thoms.) Engl. are predominantly found in the Himalayan regions of India [2].

*Bergenia* species are popularly known as ‘*Pashanbheda’* (Stone-breaker), and due to their antilithiatic and diuretic activities, these species have been traditionally used for treating kidney and urinary bladder stones in the indigenous systems of medicine in India and China [3–4]. The rhizomes, especially of *B*. *ligulata* are used as main ingredients in various Ayurvedic and Unani formulations for the treatment of urolithiasis, haemorrhoids, stomach disorders, ophthalmia, heart diseases, chronic venereal diseases, boils and blisters, leucorrhoea, piles, arthritis, epilepsy and pulmonary infections [4–5]. Numerous pharmacological activities such as antipyretic, analgesic, antioxidant, antiinflammatory, antimicrobial, antilithiatic, antiplasmodial, antitussive, antiulcer, antidiabetic, hepatoprotective, hemorrhoidal, insecticidal and diuretic properties have been reported in different species of *Bergenia* [6–12]. So far, a variety of secondary metabolites including polyphenols, quinones, steroids, carotenoids, terpenes and fatty acids have been identified from *Bergenia* species [3, 13, 14]. However, medicinal properties of *Bergenia* species are mainly due to the presence of major bioactive phenolics, e.g. bergenin (C-glycoside of 4-O-methyl gallic acid), arbutin and gallic acid, which are largely concentrated in their rhizomes [15–17]. Bergenin has been reported to exhibit antilithiatic, diuretic, antioxidant, antimicrobial, antiinflammatory, antiulcerogenic, neuroprotective, anti-HIV, antihepatotoxic and antiarrhythmic properties [15–17]. The biological activities of a plant extract depend on quantity of its bioactive markers or metabolites, which is affected by various factors such as the plant species, the time and season of harvest, climate, altitude, latitude, longitude, place of collection, age and size of a plant/plant part and phenology [18]. Previous reports indicated that variations in the quantities of bioactive phenolics of *Bergenia* species lead to variation in their medicinal activities [19]. Hence, development of an efficient analytical method to estimate the quantitative variations of bioactive phenolics in *Bergenia* species collected from diverse geographical regions of India is necessary for their quality control.

Quantitation of major bioactive markers in *B*. *ciliata*, *B*. *ligulata* and *B*. *stracheyi* using HPTLC, HPLC-PDA/UV and GC-MS methods have been carried out by several earlier workers [3–4, 8–9, 20–28]. In contrast to HPLC, HPTLC and GC-MS, the advanced ultra-high performance liquid chromatography-hybrid linear ion trap triple quadrupole mass spectrometry (UHPLC-QqQ_LIT_-MS/MS) in multiple reactions monitoring (MRM) mode ensures excellent selectivity and sensitivity for quantitative analyses in shorter duration [29]. Recently, the UHPLC-QqQ-MS, HPLC-ESI-QTOF-MS and DART-MS methods have been reported for screening and determination of active constituents of *B*. *purpurascens* and *B*. *crassifolia* (L.) Fritsch [12, 30–32].

In the present study, we have developed and validated a rapid, sensitive and specific UHPLC-QqQ_LIT_-MS/MS method in MRM mode for simultaneous determination of arbutin, bergenin, catechin, chlorogenic acid, ferulic acid, gallic acid, protocatechuic acid and syringic acid in the rhizomes of four *Bergenia* species, *viz*., *B*. *ciliata*, *B*. *ligulata*, *B*. *purpurascens* and *B*. *stracheyi*. The developed method was applied to study the quantitative variation of eight phenolics in 150 samples of four *Bergenia* species collected from different locales of the Indian Himalayas.

## Materials and methods

### Reagents, chemicals and plant materials

Acetonitrile, methanol (LC-MS grade) and formic acid (analytical grade) were procured from Fluka, Sigma-Aldrich (St. Louis, MO, USA). Ultrapure water was obtained from a Direct-Q 8 UV water purification system (EMD Millipore Corporation, Billerica, MA, USA). The analytical standards (purity ≥ 95%) of gallic acid, protocatechuic acid, chlorogenic acid and catechin were procured from Extrasynthese (Z.I. Lyon Nord, Genay Cedex, France). The analytical standards (purity ≥ 95%) of bergenin, arbutin, ferulic acid, syringic acid and vitexin were procured from Fluka, Sigma-Aldrich (St. Louis, MO, USA). The 150 rhizome samples of four *Bergenia* species were collected from various locations of India. All voucher specimens were deposited in the herbarium (LWG) of CSIR-National Botanical Research Institute, Lucknow, India. The sample details including sample code, voucher specimen number, collection location, altitude, latitude, longitude, date of collection, phenology and size of plant parts of each species are given in **S1 Table.** The species of *Bergenia* do not come under the threatened category; therefore, specific permission from the Forest Department to collect plant materials for research purpose was not required.

### Extraction and sample preparation

The dried rhizomes of 150 samples of four *Bergenia* species (approximately 10 g) were milled into powder and sieved through a 40 mesh sieve and then extracted with methanol (200 mL × 4 times) in an extractor for 36 h by maceration. The extract was filtered through Whatman filter paper and evaporated to dryness using rotary evaporator (Buchi Rotavapor-R2, Flawil, Switzerland) under reduced pressure at 40°C. To prepare 1000 μg/mL solution of each sample, dried residues (approximately 1 mg) were dissolved in appropriate amount of methanol and sonicated using ultrasonicator (Bandelin SONOREX, Berlin, Germany). The solutions were filtered through 0.22 μm syringe filter (Millex-GV, PVDF, Merck Millipore, Darmstadt, Germany). The filtrates were diluted with acetonitrile to the final working concentrations. The internal standard (vitexin) was spiked in each final working solution at a concentration of 20 ng/mL, vortexed for 30 s and 2 μL aliquot was injected into the LC-MS/MS system for analysis.

### Preparation of standard solutions

A standard stock solution (1000 μg/mL) of each reference analyte was prepared in methanol. From these stock solutions, a mixed standard stock solution (10000 ng/mL) of eight analytes was prepared in methanol. The mixed standard solution was diluted with acetonitrile to a series of concentrations within the ranges from 0.1–500 ng/mL to prepare working standard solutions. The internal standard (vitexin) was spiked in each working solution at a concentration of 20 ng/mL. The standard stock and working solutions were stored at -20°C until use and vortexed before injection.

### Instrumentation and operating conditions

The Waters ACQUITY UPLC^TM^ system (Waters, Milford, MA, USA) connected to a hybrid linear ion trap triple-quadrupole mass spectrometer (API 4000 QTRAP™ MS/MS system from AB Sciex, Concord, ON, Canada) via an electrospray (Turbo V) ion source was used in UHPLC-MS/MS analysis. The Waters ACQUITY UPLC^TM^ system was equipped with a binary solvent manager, sample manager, column oven and photodiode array detector (PDA). The control of LC-MS/MS system, data acquisition and processing was done by Analyst software (version 1.5.1, AB Sciex). The Graph Pad Prism software version 5 was used for all statistical calculations related to quantitative analysis.

#### UHPLC conditions

The chromatographic separation was carried out using an ACQUITY UPLC^TM^ BEH C_18_ column (50 mm × 2.1 mm id, 1.7 μm) maintained at 25°C. The mobile phase consisted of 0.1% (v/v) formic acid aqueous solution (A) and acetonitrile (B). The gradient elution was performed as follows: 0–1.5 min, 5–8% B; 1.5–2.5 min, 8–28% B; 2.5–3 min, 28–35% B, 3–3.5 min, 35–50% B; 3.5–4 min, 50–70% B; 4–5 min, 70–5% B and finally, the initial conditions was held for 2 min for re-equilibration. The flow rate was kept at 0.3 mL/min throughout the analysis. The sample injection volume was 2 μL.

#### MS conditions

For the quantitative determination of the target analytes, the MS instrument was operated in negative electrospray ionization mode with MRM acquisition at the unit resolution for Q1 and Q3. The optimized conditions for the electrospray source were as follows: ion spray voltage, -4200 V; curtain (CUR) gas, 20 psi; nebulizer gas (GS1) and heater gas (GS2), 50 psi; ion source temperature, 400°C; collision activated dissociation (CAD) gas, medium and the interface heater was on. The compound dependent MRM parameters including declustering potential (DP), entrance potential (EP), collision energy (CE) and cell exit potential (CXP) for precursor-to-product ion transition of each analyte were optimized by direct infusion and listed in **Table 1**. The 200 ms dwell time was used for each MRM transition.

**Table 1:** Multiple reaction monitoring (MRM) compound dependent parameters for reference analytes and internal standard.

Peak No.	t_R_ (min)	Analytes	Precursor ion (Q1)	Product ion (Q3)	DP (V)	EP (V)	CE (eV)	CXP (V)
1	0.8	Arbutin	271.0	108.0	-82	-7	-30	-9
2	1.31	Gallic acid	169.0	124.9	-59	-8	-21	-10
3	1.93	Protocatechuic acid	153.0	108.9	-64	-5	-22	-9
4	2.49	Bergenin	327.2	191.8	-83	-6	-35	-16
5	2.62	Chlorogenic acid	353.3	191.0	-60	-10	-30	-10
6	2.67	Catechin	289.1	108.9	-110	-10	-34	-9
7	2.91	Syringic acid	197.1	181.7	-57	-5	-18	-11
8	3.05	Vitexin (IS)	431.0	311.0	-92	-12	-31	-22
9	3.24	Ferulic acid	193.0	134.0	-58	-5	-23	-9

DP = declustering potential, EP = entrance potential, CE = collision energy,CXP = cell exit potential, IS = internal standard.

### Statistical analysis

Principal component analysis (PCA) was carried out to interpret the differences between 150 samples of four *Bergenia* species based on the contents of eight bioactive constituents using STATISTICA 7.0 software [33].

## Results and discussion

### Optimization of LC conditions

For achieving optimal separation of all analytes in a short analysis time, UHPLC conditions such as the column, mobile phase, gradient elution program, flow rate, injection volume and column temperature were optimized. Two different short length columns, ACQUITY UPLC^TM^ BEH C_18_ column (50 mm × 2.1 mm id, 1.7μm) and Thermo Scientific Hypersil GOLD C_18_ column (50 mm × 2.1 mm id, 1.9μm) were examined for rapid separation. An ACQUITY UPLC^TM^ BEH C_18_ column (50 mm × 2.1 mm id, 1.7 μm) was ultimately chosen for comparatively good separation efficiency and better peak shapes. Further, different gradient and mobile phase systems (water–methanol, water–acetonitrile, 0.1% (v/v) formic acid aqueous solution–methanol and 0.1% (v/v) formic acid aqueous solution–acetonitrile) were compared at different column temperatures (20°C, 25°C, 35°C and 45°C) and flow rates (0.2, 0.25, 0.3, 0.35 and 0.4 mL/min). Optimization results showed that mobile phase system composed of 0.1% (v/v) formic acid aqueous solution and acetonitrile afforded optimum separation and ionization at a flow rate of 0.3 mL/min and 25°C column temperature within 5 min. The developed method is rapid as compared to previously reported HPLC, UHPLC-QqQ-MS and HPLC-QTOF-MS methods [23, 27, 32] for screening and quantitation of phenolics, having chromatographic run time of 8.5–50 min.

### Optimization of MS conditions

**Fig 1:**
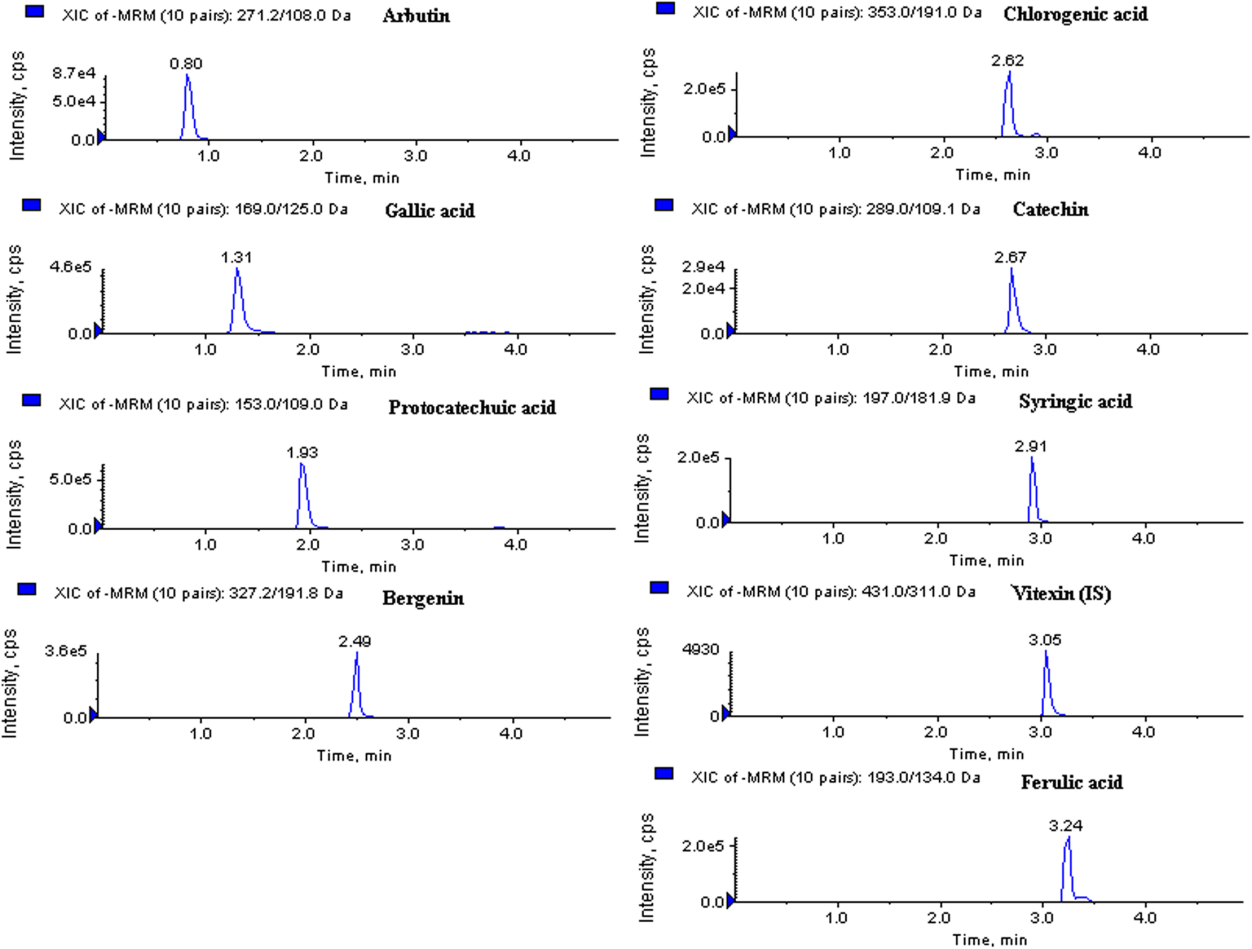
UHPLC-MRM extracted ion chromatogram of analytes and internal standard.

The mass spectrometric conditions were optimized by direct infusion of 50 ng/mL solution of each targeted analyte into mass spectrometer at a flow rate of 10 μL/min using a Harvard ‘22’ syringe pump (Harvard Apparatus, South Natick, MA, USA). MS spectra were recorded in both positive and negative ionization mode. Finally, negative ionization mode was selected due to the high signal sensitivity of all target analytes in that mode. In order to achieve most abundant, specific and stable MRM transition for each analyte, the compounds dependent MRM parameters (DP, EP, CE and CXP) and source parameters (curtain gas, GS1, GS2 and ion source temperature) were optimized (**Table 1)**. The optimized UHPLC-MS/MS method in MRM acquisition mode was applied to quantify eight bioactive phenolics in rhizomes of the four *Bergenia* species. UHPLC-MRM extracted ion chromatogram of analytes, and internal standard is presented in **Fig 1**.

### Identification of targeted analytes

The identity of target analytes in the samples was confirmed by the comparison of their retention times and MS/MS spectra with authentic standards. The dominant product ion of each target analyte was selected for MRM transition. The ESI-MS spectra generated deprotonated molecule [M-H]^-^ for phenolics in the negative ion mode. The MS/MS spectra of the eight analytes, internal standard and the fragmentation scheme selected for MRM transition are shown in **S1–S3 Figs.** The chemical structures of all investigated compounds were confirmed by their diagnostic fragment ions such as [Y_0_-H]^-•^, [M-H-CO_2_]^-^, [M-H-C_4_H_8_O_4_-CH_3_]^-^, [M-H-C_9_H_6_O_3_]^-^, [M-H-C_9_H_8_O_4_]^-^, [M-H-CH_3_]^-^, ^0,2^X^-^ and [M-H-CO_2_-CH_3_]^-^ [34–38]. The developed UHPLC-MS/MS method is more specific than the earlier reported ones [12, 23, 27, 30] as the targeted active phenolics were confirmed by their [M-H]^-^ ions and diagnostic fragment ions in the samples investigated during the present study.

### Analytical method validation

The developed UHPLC-MS/MS method was validated in terms of linearity, limit of detection (LOD), limit of quantitation (LOQ), precision, stability and accuracy in accordance with the International Conference on Harmonization (ICH, Q2R1) guidelines.

#### Linearity, LOD and LOQ

The linearity of the developed method was assessed at seven concentration levels from 0.1–500 ng/mL. The calibration curves were prepared by plotting the analytes-to-IS peak area ratios against the corresponding concentrations. The LOD and LOQ were determined by calibration curve method using following equations: **LOD = (3.3×S**_***xy***_**)/S**_**a**_ and **LOQ = (10×S**_***xy***_**)/S**_**a**_, where S_xy_ is the residual standard deviation of the regression line and S_a_ is the slope of a calibration curve (**Table 2)**. The calibration curves of all analytes exhibited good linearity with correlation coefficients (*r*^*2*^) ranging from 0.9991–1.0000 within the test ranges. The LOD for eight analytes was ranged from 0.05–0.78 ng/mL and LOQ from 0.14–2.36 ng/mL. The previously reported LOD and LOQ values for these phenolics ranged from 0.04 to 10.7 μg/mL by HPLC and HPTLC methods. However, in the present study the LOD and LOQ values for all analytes are ≤ 2.36 ng/mL, thus showing higher sensitivity than the earlier reports [8, 19, 21, 23–24, 27–28, 39].

**Table 2:** Method validation parameters for eight reference analytes.

	Analytes							
Parameters	Arbutin	Gallic acid	Protocatechuic acid	Bergenin	Chlorogenic acid	Catechin	Syringic acid	Ferulic acid
Regression equation	*y* = 0.221*x* - 0.018	*y* = 1.484*x* - 0.001	*y* = 1.863*x* - 0.185	*y* = 0.694*x* - 0.011	*y* = 0.726*x* - 0.114	*y* = 0.064*x* + 0.048	*y* = 0.371*x* + 0.021	*y* = 0.560*x* - 0.032
Correlation coefficient (*r*2)	0.9996	0.9991	0.9994	1.0000	0.9995	0.9994	0.9993	0.9998
Linearity range (ng/mL)	10–300	10–300	10–300	10–300	10–300	25–500	10–300	10–300
LOD (ng/mL)	0.36	0.57	0.48	0.05	0.43	0.78	0.50	0.29
LOQ (ng/mL)	1.09	1.71	1.45	0.14	1.30	2.36	1.53	0.87
Precision RSD % (Intra-day, *n* = 6)	1.05	1.17	0.27	0.65	1.19	0.95	0.74	1.24
Precision RSD % (Inter-day, *n* = 6)	1.87	1.86	1.27	1.25	1.50	1.12	1.33	1.87
Stability RSD % (*n* = 5)	2.39	2.55	2.52	1.90	2.36	1.63	2.51	2.26
Recovery (*n* = 3) Mean	97.04	98.64	97.29	100.12	100.21	99.51	102.11	95.16
RSD %	0.96	1.11	1.83	1.21	1.18	1.07	1.43	1.75

*y* = peak area of analyte

*x* = concentration of analyte

#### Precision, stability and accuracy

The intra-day and inter-day variations were selected to determine the precision of the established method. For intra-day and inter-day variability test, three concentration levels of working standard solutions were analyzed in six replicates within a single day and by repeating the experiments on three consecutive days. Variations of the peak area were expressed by percentage relative standard deviations (RSD) and taken as the measures of precision. The overall intra-day and inter-day precisions were not more than 1.87%. For stability test, sample solutions stored at room temperature were analyzed by replicate injections at 0, 2, 4, 8, 12 and 24 h. The RSD values of stability of the eight analytes were ≤ 2.55% (**Table 2**).

The recovery experiment was used to assess the accuracy of the developed method. It was performed by adding known amounts of eight analytical standards at low, medium and high levels into samples. The spiked samples were then analyzed at each level in triplicate. The average recovery of each analyte was estimated using the following equation:

Recovery(%)=(observedamount–originalamount)/spikedamount×100%.

The average recoveries of all analytes were ranged from 95.16–102.11% with RSD ≤ 1.83%, demonstrated that the developed method is accurate (**Table 2**).

The results of method validation assay showed that the developed method fulfilled all criteria of a validated method as per ICH guidelines with the acceptable ranges of linearity (r^2^ ≤ 0.9991), LOD and LOQ (≤ 2.36 ng/mL), precision and accuracy (RSD ≤ 1.87%) [40].

### Method application

**Fig 2:**
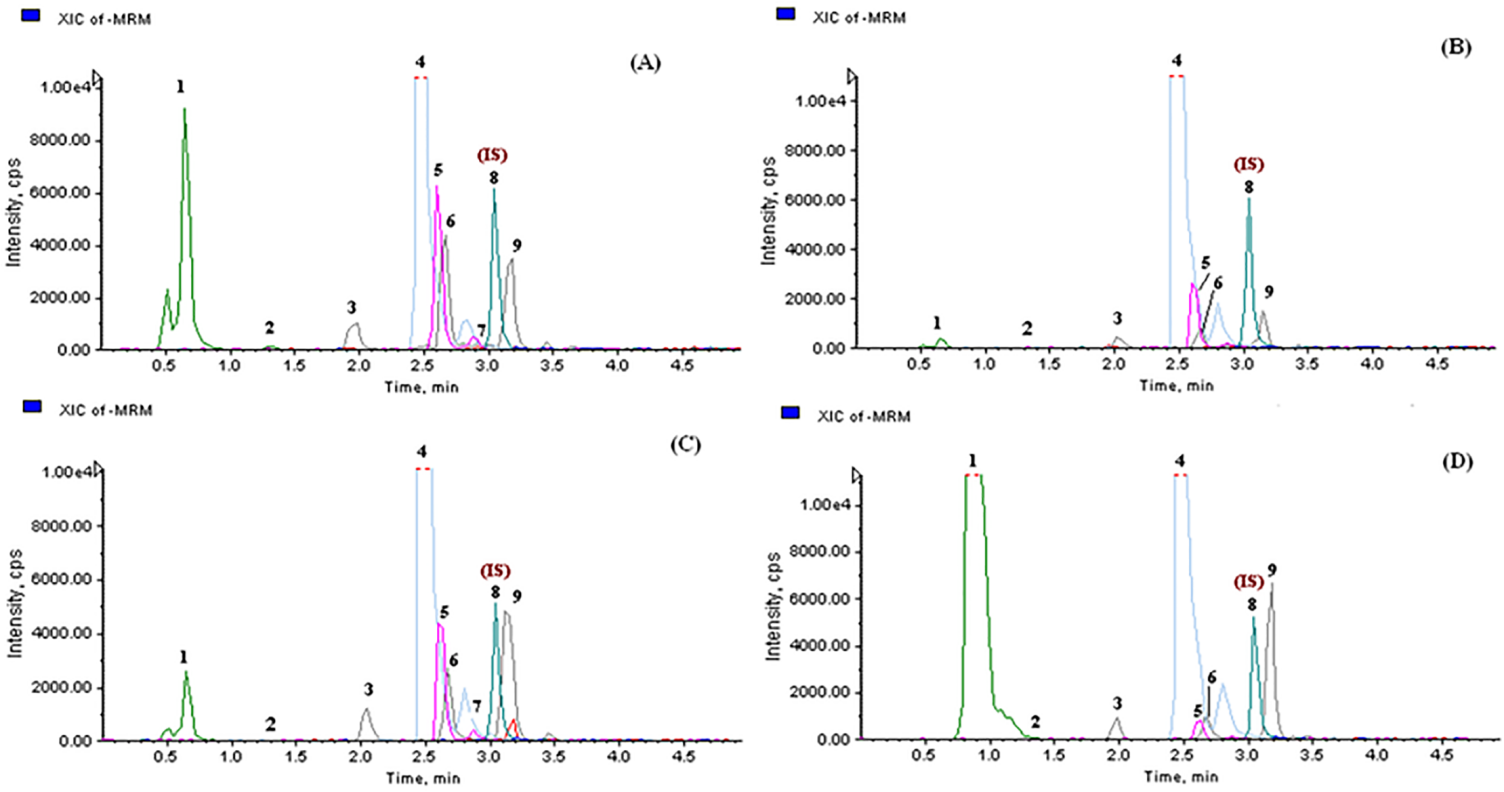
UHPLC-MRM extracted ion chromatograms of *Bergenia* species: **(A**) *B*. *ciliata*, **(B)** *B*. *ligulata*, **(C)** *B*. *purpurascens*, **(D)** *B*. *stracheyi*.

The newly developed UHPLC-MS/MS method was applied to estimate the quantitative variation of eight bioactive phenolics in 150 samples of four *Bergenia* species that included 103 samples of *B*. *ciliata*, 24 samples of *B*. *ligulata*, 7 samples of *B*. *purpurascens* and 16 samples of *B*. *stracheyi*. The content of eight analytes was calculated with internal standard method based on their corresponding calibration curves (**Table 3**). UHPLC-MRM extracted ion chromatograms of investigated *Bergenia* species are shown in **Fig 2.**

**Table 3:** Content (mg/g) of eight phenolics in 150 samples of four *Bergenia* species.

Sample code	Analytes (mg/g) mean (±SD, *n* = 3)																Total
	Arbutin		Gallic acid		Protocatechuic acid		Bergenin		Chlorogenic acid		Catechin		Syringic acid		Ferulic acid		
BS-1	0.78	±0.002	0.55	±0.01	0.321	±0.004	9.68	±0.013	2.2	±0.002	2.24	±0.002	0.21	±0.001	0.23	±0.002	16.3
BS-2	0.31	±0.01	nd	0	0.337	±0.001	11.9	±0.02	2.6	±0.016	1.55	±0.002	0.13	±0.002	0.15	±0.0015	17
BS-3	0.289	±0.02	0.53	±0.001	0.286	±0.003	12	±0.122	1.5	±0.001	4.44	±0.003	0.1	±0.001	0.145	±0.0015	19.3
BS-4	0.335	±0.013	0.22	±0.004	0.247	±0.001	6.39	±0.003	2.4	±0.012	4.29	±0.002	nd	0	0.163	±0.0003	14
BS-5	0.552	±0.05	0.77	±0.003	0.251	±0.004	9.35	±0.002	24	±0.019	14.8	±0.015	0.1	±0.001	0.148	±0.001	49.6
BS-6	1.05	±0.001	1.05	±0.002	0.412	±0.001	10.2	±0.01	5.9	±0.006	11.9	±0.01	0.25	±0.003	0.168	±0.001	30.9
BS-7	0.53	±0.002	0.11	±0.001	0.276	±0.013	8.77	±0.001	3.5	±0.05	9.89	±0.001	0.09	±0.001	0.119	±0.0015	23.3
BS-8	0.57	±0.001	0.24	±0.006	0.248	±0.002	19.2	±0.002	2.4	±0.001	8.99	±0.002	0.07	±0.005	0.199	±0.0006	31.9
BS-9	0.747	±0.006	0.51	±0.002	0.247	±0.002	22.9	±0.006	3.5	±0.04	11.7	±0.007	0.07	±0.001	0.181	±0.004	39.8
BS-10	0.554	±0.004	1.13	±0.015	0.249	±0.011	12.6	±0.007	3.2	±0.002	6.8	±0.01	0.08	±0.004	0.134	±0.001	24.7
BS-11	0.489	±0.001	0.55	±0.001	0.238	±0.001	19.6	±0.007	5.1	±0.011	4.39	±0.001	0.22	±0.001	0.143	±0.001	30.7
BS-12	0.425	±0.002	0.56	±0.002	0.289	±0.001	17.1	±0.01	4	±0.052	3.87	±0.001	0.08	±0.002	0.162	±0.001	26.5
BS-13	0.383	±0.001	0.14	±0.001	0.266	±0.013	14.3	±0.01	5.8	±0.001	13.6	±0.01	0.05	±0.002	0.165	±0.002	34.7
BS-14	0.781	±0.005	0.83	±0.001	0.314	±0.001	12.9	±0.015	4.7	±0.023	12.1	±0.002	0.06	±0.001	0.154	±0.001	31.8
BS-15	1.05	±0.002	0.7	±0.002	0.388	±0.002	21.6	±0.01	4.8	±0.001	5.38	±0.001	0.1	±0.001	0.146	±0.001	34.2
BS-16	0.87	±0.003	0.78	±0.001	0.37	±0.002	8.67	±0.001	4.1	±0.002	5.76	±0.002	0.04	±0.005	0.133	±0.001	20.7
BS-17	0.978	±0.02	0.76	±0.003	0.325	±0.013	11.6	±0.01	2.2	±0.003	1.6	±0.007	0	±0.001	0.178	±0.0015	17.6
BS-18	1.31	±0.018	0.38	±0.002	0.242	±0.001	12.4	±0.01	3.7	±0.001	9.66	±0.047	0.03	±0.001	0.241	±0.001	27.9
BS-19	0.343	±0.001	nd	0	0.283	±0.001	12.7	±0.02	7.8	±0.007	7.91	±0.001	0.11	±0.003	0.155	±0.001	29.3
BS-20	0.348	±0.003	0.35	±0.004	0.352	±0.003	26.6	±0.01	5.5	±0.016	19.2	±0.01	0.18	±0.001	0.2	±0.0115	52.7
BS-21	0.476	±0.002	0.14	±0.002	0.25	±0.004	15.3	±0.01	1.5	±0.002	5.54	±0.001	0.11	±0.005	0.235	±0.0038	23.6
BS-22	1.94	±0.007	0.85	±0.002	0.278	±0.002	21.8	±0.01	7.9	±0.001	11.8	±0.021	0.02	±0.002	0.175	±0.001	44.7
BS-23	1.68	±0.002	0.8	±0.007	0.284	±0.001	26.6	±0.006	4.3	±0.008	10.6	±0.01	0.06	±0.002	0.163	±0.0026	44.5
BS-24	0.297	±0.003	0.25	±0.001	0.262	±0.003	14.3	±0.003	3.4	±0.022	6.26	±0.002	0.05	±0.003	0.166	±0.0015	25
BS-25	0.715	±0.012	0.35	±0.001	0.239	±0.001	11	±0.03	2.2	±0.023	4.2	±0.01	0.12	±0.001	0.187	±0.001	19
BS-26	1.19	±0.002	nd	0	0.257	±0.001	7.88	±0.001	6.3	±0.067	5.89	±0.001	0.08	±0.001	0.215	±0.001	21.8
BS-27	1.23	±0.006	0.48	±0.004	0.25	±0.001	16.9	±0.01	5	±0.002	7.93	±0.001	0.07	±0.001	0.177	±0.002	32
BS-28	2.14	±0.02	0.89	±0.001	0.396	±0.001	19.2	±0.01	2.9	±0.098	5.01	±0.01	0.16	±0.002	0.16	±0.001	30.9
BS-29	1.9	±0.001	0.73	±0.005	0.344	±0.001	18.6	±0.02	5.5	±0.002	10.8	±0.002	0.1	±0.002	0.282	±0.001	38.3
BS-30	0.656	±0.007	0.31	±0.001	0.22	±0.002	10	±0.006	3.1	±0.043	3.49	±0.001	0.03	±0.001	0.166	±0.001	17.9
BS-31	0.442	±0.001	0.18	±0.002	0.226	±0.001	4.95	±0.002	0.9	±0.001	1.44	±0.002	nd	0	0.171	±0.001	8.34
BS-32	0.402	±0.008	0.91	±0.013	0.324	±0.001	8.3	±0.01	1.7	±0.006	4.8	±0.01	0.04	±0.003	0.147	±0.001	16.6
BS-33	0.442	±0.002	1.13	±0.017	0.28	±0.003	14.9	±0.01	1.7	±0.009	1.57	±0.002	0.05	±0.001	0.3	±0.01	20.3
BS-34	0.527	±0.007	0.12	±0.001	0.263	±0.001	9.95	±0.002	4.3	±0.001	6.27	±0.001	0.04	±0.001	0.147	±0.001	21.6
BS-35	0.433	±0.001	0.37	±0.001	0.277	±0.002	7.05	±0.001	1.8	±0.001	5.32	±0.001	0.07	±0.004	0.156	±0.002	15.5
BS-36	0.835	±0.008	0.3	±0.020	0.427	±0.005	8.5	±0.02	5.7	±0.015	5.05	±0.002	0.05	±0.002	0.144	±0.002	21
BS-37	0.528	±0.02	1.22	±0.005	0.28	±0.002	9.96	±0.002	1.7	±0.02	5.03	±0.001	0.09	±0.001	0.209	±0.001	19
BS-38	0.611	±0.012	0.05	±0.001	0.322	±0.001	9.53	±0.001	2.6	±0.002	3.86	±0.01	nd	0	0.264	±0.001	17.2
BS-39	0.511	±0.001	0.91	±0.002	0.285	±0.03	12.6	±0.02	3.1	±0.001	5.64	±0.001	nd	0	0.225	±0.0005	23.3
BS-40	0.614	±0.03	0.67	±0.001	0.262	±0.001	8.8	±0.01	5	±0.008	15	±0.1	0.08	±0.001	0.186	±0.001	30.6
BS-41	1.38	±0.002	0.72	±0.001	0.229	±0.006	5.65	±0.02	7.5	±0.02	12.5	±0.02	0.07	±0.004	0.2	±0.0058	28.2
BS-42	0.928	±0.001	nd	0	0.258	±0.001	12.9	±0.01	11	±0.02	30.2	±0.01	nd	0	0.216	±0.001	55.4
BS-43	0.991	±0.005	0.2	±0.001	0.252	±0.005	17.3	±0.01	5.3	±0.032	15	±0.02	nd	0	0.171	±0.0021	39.2
BS-44	0.935	±0.03	0.47	±0.001	0.311	±0.01	15.3	±0.056	6.9	±0.025	9.56	±0.05	0.05	±0.002	0.157	±0.0001	33.6
BS-45	1.42	±0.015	1.01	±0.011	0.259	±0.001	20.9	±0.013	4.5	±0.034	17.8	±0.032	0.31	±0.001	0.174	±0.0006	46.4
BS-46	0.785	±0.001	0.45	±0.001	0.249	±0.002	30.4	±0.02	12	±0.02	22.4	±0.02	nd	0	0.2	±0.01	66
BS-47	2.36	±0.002	0.95	±0.001	0.272	±0.002	16.1	±0.208	9.1	±0.002	16	±0.064	0.03	±0.002	0.146	±0.0001	44.9
BS-48	0.533	±0.004	0.82	±0.002	0.262	±0.001	27.8	±0.02	2.4	±0.001	7.12	±0.001	nd	0	0.162	±0.002	39.1
BS-49	1.01	±0.001	0.96	±0.001	0.328	±0.023	26	±0.1	2.5	±0.017	10.2	±0.01	0.02	±0.001	0.137	±0.003	41.1
BS-50	0.809	±0.001	1.47	±0.001	0.426	±0.006	23.4	±0.02	1.4	±0.02	8.22	±0.001	0	±0.0002	0.171	±0.0006	35.9
BS-51	0.605	±0.006	0.44	±0.002	0.322	±0.011	32.1	±0	3.2	±0.05	6.46	±0.013	nd	0	0.181	±0.0006	43.4
BS-52	0.225	±0.001	0.28	±0.001	0.244	±0.001	31.1	±0.182	1.4	±0.02	25.5	±0.01	0.06	±0.001	0.138	±0.0007	58.9
BS-53	1.31	±0.008	0.74	±0.001	0.312	±0.065	28.8	±0.15	2.5	±0.011	10	±0.033	0.04	±0.003	0.161	±0.001	43.9
BS-54	1.28	±0.002	0.47	±0.001	0.28	±0.002	22.4	±0.07	1.3	±0.001	12.3	±0.02	nd	0	0.174	±0.001	38.2
BS-55	3.26	±0.001	1.55	±0.02	0.28	±0.019	26.2	±0.02	1.6	±0.006	21.2	±0.01	nd	0	0.249	±0.0006	54.3
BS-56	3.47	±0.015	0.74	±0.001	0.258	±0.001	39.5	±0.09	1	±0.002	15.5	±0.021	nd	0	0.157	±0.0015	60.6
BS-57	2.03	±0.002	0.78	±0.001	0.385	±0.035	27.2	±0.01	1.4	±0.042	9.12	±0.003	0.05	±0.001	0.18	±0.002	41.2
BS-58	20.1	±0.05	0.55	±0.016	0.318	±0.006	39.2	±0.086	3.3	±0.001	32.4	±0.032	0.04	±0.0002	0.209	±0.0007	96.1
BS-59	3.74	±0.012	0.45	±0.001	0.385	±0.002	11.9	±0.01	1.3	±0.013	13.9	±0.002	nd	0	0.243	±0.001	31.9
BS-60	2.25	±0.001	1.06	±0.001	0.323	±0.014	24.7	±0.017	4.6	±0.041	24.3	±0.019	nd	0	0.204	±0.001	57.4
BS-61	1.01	±0.015	1.74	±0.001	0.379	±0.001	33.2	±0.03	1.9	±0.002	18.1	±0.01	0.06	±0.0001	0.152	±0.0006	56.6
BS-62	8.4	±0.002	2.08	±0.002	0.298	±0.002	27.7	±0.02	2.7	±0.015	11	±0.02	0.23	±0.002	0.228	±0.002	52.6
BS-63	0.215	±0.006	0.43	±0.002	0.224	±0.001	22.4	±0.03	4.4	±0.001	10.3	±0.012	0.13	±0.011	0.169	±0.0006	38.3
BS-64	0.223	±0.02	0.87	±0.002	0.224	±0.001	34.3	±0.012	9.8	±0.002	26.6	±0.1	nd	0	0.185	±0.0015	72.2
BS-65	0.194	±0.0006	1.22	±0.001	0.354	±0.001	31.3	±0.02	7.4	±0.002	28	±0.012	0.09	±0.001	0.179	±0.0006	68.7
BS-66	0.218	±0.012	0.2	±0.001	0.226	±0.057	35.3	±0.02	2.9	±0.003	37.3	±0.02	nd	0	0.279	±0.001	76.5
BS-67	0.129	±0.007	0.77	±0.002	0.366	±0.063	27.2	±0.01	18	±0.001	35.4	±0.1	0.11	±0.001	0.182	±0.0005	81.9
BS-68	0.158	±0.0006	0.36	±0.001	0.243	±0.001	15.8	±0.012	1.9	±0.006	11.2	±0.02	nd	0	0.215	±0.0006	29.9
BS-69	0.134	±0.004	0.21	±0.006	0.238	±0.012	21.8	±0.012	7.6	±0.02	24.5	±0.019	0.08	±0.003	0.178	±0.0025	54.7
BS-70	0.16	±0.006	0.05	±0.002	0.362	±0.003	28.5	±0.02	4.8	±0.002	15.1	±0.017	0.05	±0.0003	0.163	±0.001	49.1
BS-71	0.155	±0.005	0.11	±0.002	0.259	±0.016	29.2	±0.006	13	±0.019	38.3	±0.029	nd	0	0.231	±0.001	81.2
BS-72	2.93	±0.006	1.74	±0.001	0.455	±0.003	30.8	±0.01	1.3	±0.001	nd	0	0.8	±0.001	0.288	±0.001	38.3
BS-73	0.591	±0.002	2.12	±0.001	0.412	±0.001	26.3	±0.012	7	±0.01	0.58	±0.02	0.04	±0.0003	0.205	±0.001	37.3
BS-74	0.749	±0.03	2	±0.012	0.332	±0.002	2.39	±0.02	11	±0.02	3.34	±0.017	0.05	±0.001	0.196	±0.0006	19.9
BS-75	0.473	±0.006	0.93	±0.001	0.299	±0.001	17.1	±0.02	2.4	±0.001	nd	0	0.19	±0.001	0.152	±0.002	21.5
BS-76	1.71	±0.003	2.11	±0.100	0.457	±0.002	38	±0.002	1	±0.02	nd	0	0.68	±0.002	0.239	±0.001	44.2
BS-77	0.461	±0.002	0.96	±0.001	0.258	±0.001	4.9	±0.1	2.1	±0.02	1.34	±0.011	0.08	±0.001	0.175	±0.002	10.3
BS-78	1.39	±0.006	0.99	±0.016	0.231	±0.023	24.2	±0.2	0.8	±0.001	0.06	±0.0003	0.05	±0.002	0.242	±0.001	28
BS-79	1.41	±0.005	1.12	±0.001	0.317	±0.001	33.4	±0.002	0.8	±0.007	2.79	±0.007	nd	0	0.185	±0.0012	40
BS-80	0.276	±0.001	0.89	±0.001	0.404	±0.016	15	±0.01	0.7	±0.011	15.2	±0.001	nd	0	0.17	±0.0008	32.6
BS-81	0.657	±0.012	0.69	±0.019	0.23	±0.002	7.29	±0.02	2.3	±0.001	6.39	±0.017	0.23	±0.005	0.154	±0.002	17.9
BS-82	0.521	±0.002	0.86	±0.001	0.22	±0.004	6.15	±0.012	0.9	±0.002	12.2	±0.02	nd	0	0.182	±0.002	21.1
BS-83	0.339	±0.002	0.84	±0.012	0.356	±0.002	15.4	±0.01	1.9	±0.053	14.7	±0.04	0.01	±0.001	0.181	±0.001	33.7
BS-84	2.61	±0.001	4.56	±0.003	0.327	±0.001	13	±0.002	8.3	±0.002	4.77	±0.002	nd	0	0.118	±0.0006	33.7
BS-85	0.681	±0.001	1.12	±0.001	0.419	±0.002	15.4	±0.012	2.8	±0.051	7.97	±0.01	0.11	±0.001	0.135	±0.0006	28.6
BS-86	0.427	±0.002	4.8	±0.002	0.556	±0.004	35.2	±0.2	1.6	±0.08	0.1	±0.002	nd	0	0.168	±0.0005	42.8
BS-87	0.203	±0.001	0.36	±0.002	0.25	±0.001	39.6	±0.02	2	±0.012	10.6	±0.011	0.02	±0.003	0.206	±0.0006	53.2
BS-88	1.05	±0.002	2.67	±0.012	0.378	±0.011	55	±0.01	2.5	±0.002	6.6	±0.01	0.1	±0.0001	0.173	±0.001	68.4
BS-89	0.28	±0.002	0.18	±0.001	0.258	±0.001	14.8	±0.001	3.3	±0.001	5.97	±0.02	nd	0	0.136	±0.001	24.9
BS-90	0.157	±0.013	0.84	±0.005	0.249	±0.002	6.84	±0.2	4.8	±0.001	0.77	±0.002	nd	0	0.161	±0.002	13.8
BS-91	0.215	±0.001	0.73	±0.002	0.252	±0.017	9.57	±0.02	5.1	±0.003	3.8	±0.001	nd	0	0.122	±0.002	19.8
BS-92	0.211	±0.001	1.15	±0.003	0.349	±0.001	22	±0.001	6.2	±0.01	22.7	±0.002	nd	0	0.122	±0.0006	52.7
BS-93	0.787	±0.002	1.12	±0.001	0.344	±0.023	35.9	±0.006	7.3	±0.004	6.56	±0.02	nd	0	0.343	±0.001	52.3
BS-94	1.11	±0.001	0.76	±0.005	0.278	±0.001	20.7	±0.001	11	±0.001	8.42	±0.02	0.07	±0.0003	0.19	±0.002	42.7
BS-95	0.38	±0.006	0.44	±0.002	0.239	±0.002	8.28	±0.01	2.4	±0.019	5.39	±0.001	nd	0	0.158	±0.003	17.3
BS-96	1.37	±0.005	1.95	±0.001	0.717	±0.062	39.8	±0.03	1.2	±0.01	1.42	±0.006	nd	0	0.246	±0.0015	46.7
BS-97	0.241	±0.001	0.96	±0.001	0.239	±0.001	22.3	±0.001	4.9	±0.006	5.33	±0.023	nd	0	0.109	±0.002	34.1
BS-98	0.268	±0.002	1.56	±0.002	0.286	±0.032	31.9	±0.001	0.9	±0.01	9.8	±0.001	nd	0	0.222	±0.0006	44.9
BS-99	0.168	±0.001	1.65	±0.001	0.273	±0.001	18.3	±0.01	1.9	±0.012	0.83	±0.001	nd	0	0.183	±0.001	23.3
BS-100	0.365	±0.006	6.29	±0.002	0.477	±0.013	33.4	±0.02	0.4	±0.002	0.45	±0.001	0.06	±0.0004	0.147	±0.0006	41.6
BS-101	0.354	±0.002	2.81	±0.004	0.492	±0.001	15.6	±0.02	4.6	±0.011	1.11	±0.002	nd	0	0.218	±0.0025	25.1
BS-102	0.26	±0.002	3.03	±0.010	0.396	±0.003	72.1	±0.012	1.4	±0.003	2.22	±0.002	0.15	±0.001	0.369	±0.002	80
BS-103	0.416	±0.006	1.19	±0.001	0.385	±0.055	11.2	±0.001	4.4	±0.015	4.2	±0.001	nd	0	0.141	±0.001	22
BS-104	0.532	±0.002	0.11	±0.025	0.251	±0.06	56.5	±0.01	1.9	±0.002	2.37	±0.001	0	±0.001	0.242	±0.0006	61.9
BS-105	0.187	±0.001	0.44	±0.01	0.383	±0.001	225	±0.006	0.4	±0.002	nd	0	nd	0	0.177	±0.0006	227
BS-106	0.267	±0.006	1.38	±0.002	0.259	±0.002	72.8	±0.02	0.5	±0.002	nd	0	nd	0	0.162	±0.002	75.4
BS-107	0.206	±0.001	0.39	±0.001	0.233	±0.025	52.1	±0.006	1.6	±0.015	9.97	±0.01	nd	0	0.15	±0.002	64.6
BS-108	0.17	±0.001	0.93	±0.001	0.256	±0.001	136	±0.01	0.4	±0.007	3.16	±0.1	0.02	±0.001	0.533	±0.001	141
BS-109	0.388	±0.003	0.54	±0.002	0.294	±0.001	28.5	±0.02	1.6	±0.002	4.13	±0.01	0.05	±0.001	0.22	±0.0006	35.8
BS-110	0.172	±0.010	3.79	±0.002	0.242	±0.002	61.5	±0.018	1.7	±0.001	5.47	±0.02	0.04	±0.001	0.145	±0.0006	73.1
BS-111	0.301	±0.020	0.94	±0.001	0.258	±0.051	67.2	±0.059	0.5	±0.02	nd	0	0.02	±0.001	0.178	±0.0006	69.4
BS-112	0.636	±0.002	1.06	±0.001	0.375	±0.011	55.6	±0.089	1.2	±0.001	4.38	±0.001	0.22	±0.002	0.208	±0.0006	63.7
BS-113	0.168	±0.001	2.74	±0.01	0.287	±0.002	50.4	±0.01	0.5	±0.012	0.78	±0.002	0.3	±0.011	0.387	±0.0002	55.6
BS-114	nd	0	0.84	±0.001	0.245	±0.001	243	±0.001	0.4	±0.01	nd	0	nd	0	0.206	±0.001	245
BS-115	0.647	±0.010	0.17	0.000	0.269	±0.001	40.3	±0.02	7.1	±0.001	21.2	±0.02	0.07	±0.0003	0.123	±0.002	69.9
BS-116	0.696	±0.001	3.1	±0.001	0.325	±0.002	40	±0.001	1.5	±0.002	3.03	±0.05	nd	0	0.16	±0.002	48.8
BS-117	0.258	±0.010	1.21	±0.001	0.291	±0.001	80	±0.001	2.4	±0.001	5.37	±0.01	0.03	±0.0001	0.169	±0.0006	89.7
BS-118	0.72	±0.020	0.84	±0.005	0.251	±0.015	59.4	±0.01	2.2	±0.003	8.58	±0.02	0.02	±0.00003	0.259	±0.0006	72.3
BS-119	0.266	±0.010	2.89	±0.002	0.442	±0.001	79.7	±0.02	2.8	±0.001	4.99	±0.021	0.08	±0.001	0.164	±0.0007	91.3
BS-120	0.52	±0.020	1.5	±0.003	0.231	±0.062	48.3	±0.01	0.4	±0.001	0.37	±0.001	nd	0	0.216	±0.001	51.6
BS-121	1.24	±0.100	2.29	±0.015	0.316	±0.001	56.9	±0.02	1.8	±0.011	2.9	±0.015	nd	0	0.19	±0.0004	65.6
BS-122	0.368	±0.001	3.67	±0.002	0.28	±0.006	69.7	±0.01	1.5	±0.023	1.78	±0.02	0.16	±0.004	0.286	±0.0006	77.8
BS-123	0.565	±0.010	0.37	±0.006	0.281	±0.02	34.8	±0.001	6.7	±0.016	7.75	±0.02	nd	0	0.242	±0.0005	50.7
BS-124	0.343	±0.019	0.52	±0.003	0.243	±0.001	27.6	±0.01	2.4	±0.001	24.4	±0.001	0.05	±0.001	0.164	±0.001	55.7
BS-125	0.145	±0.010	0.86	±0.001	0.279	±0.002	94.7	±0.001	0.5	±0.004	1.88	±0.018	0.06	±0.0002	0.155	±0.001	98.6
BS-126	0.173	±0.001	0.36	±0.002	0.353	±0.016	133	±0.067	0.4	±0.002	0.4	±0.001	nd	0	0.23	±0.0005	135
BS-127	0.767	±0.003	23.5	±0.001	0.284	±0.022	41.1	±0.001	2.9	±0.02	3.54	±0.019	nd	0	0.141	±0.001	72.3
BS-128	6.32	±0.001	1.23	±0.011	0.25	±0.001	49.6	±0.02	1.7	±0.001	24	±0.025	0.6	±0.001	0.153	±0.001	83.9
BS-129	5.3	±0.007	1.07	±0.001	0.272	±0.067	41.6	±0.001	1.6	±0.012	11.3	±0.002	0.2	±0.012	0.178	±0.001	61.5
BS-130	8.48	±0.001	0.08	±0.003	0.287	±0.001	47.2	±0.001	2.2	±0.01	20	±0.031	0.29	±0.001	0.214	±0.0006	78.7
BS-131	8.59	±0.002	1.05	±0.001	0.282	±0.033	59.6	±0.09	2.1	±0.013	33.1	±0.025	0.69	±0.001	0.196	±0.0001	106
BS-132	8.6	±0.002	0.72	±0.002	0.343	±0.001	62.9	±0.001	3	±0.006	28.6	±0.001	0.52	±0.001	0.203	±0.001	105
BS-133	11.2	±0.001	1.27	±0.001	0.414	±0.002	53.3	±0.01	3.7	±0.001	23.9	±0.053	0.33	±0.001	0.174	±0.0004	94.3
BS-134	6.68	±0.002	0.89	±0.011	0.306	±0.007	64.8	±0.02	3.2	±0.003	15.7	±0.002	0.75	±0.001	0.225	±0.001	92.5
BS-135	77.7	±0.001	2.41	±0.006	0.316	±0.002	80	±0.02	0.8	±0.001	7.67	±0.001	nd	0	0.208	±0.0015	169
BS-136	63.9	±0.036	1.02	±0.001	0.257	±0.001	55.2	±1.06	4.1	±0.001	6.72	±0.012	0.06	±0.0002	0.166	±0.001	131
BS-137	55	±0.001	4.21	±0.002	0.274	±0.008	66.9	±0.15	0.8	±0.015	8.81	±0.02	nd	0	0.262	±0.001	136
BS-138	27.4	±0.002	0.1	±0.015	0.425	±0.001	60.7	±0.001	1.1	±0.001	14	±0.002	nd	0	0.174	±0.002	104
BS-139	21.4	±0.001	nd	0	0.302	±0.012	58.3	±0.25	0.6	±0.002	10.1	±0.005	nd	0	0.234	±0.0006	90.9
BS-140	6.77	±0.005	0.89	±0.002	0.238	±0.001	49.4	±0.01	0.6	±0.001	6.1	±0.002	nd	0	0.157	±0.0005	64.1
BS-141	15.9	±0.007	0.43	±0.005	0.304	±0.003	55.8	±0.095	0.5	±0.002	11.9	±0.045	0.01	±0.002	0.141	±0.0006	85
BS-142	16.2	±0.001	0.51	±0.02	0.326	±0.001	44.1	±0.02	0.6	±0.001	16.3	±0.001	0.03	±0.0003	0.235	±0.0006	78.3
BS-143	50.4	±0.018	1.04	±0.012	0.259	±0.01	52.1	±0.53	0.9	±0.003	7.23	±0.019	0.05	±0.0001	0.165	±0.001	112
BS-144	36.6	±0.001	0.18	±0.01	0.281	±0.001	67.6	±0.01	0.6	±0.002	1.32	±0.001	0	±0.001	0.199	±0.002	107
BS-145	15.8	±0.006	7.35	±0.001	0.275	±0.05	38.4	±0.001	0.6	±0.001	3.99	±0.057	0.02	±0.0001	0.121	±0.002	66.6
BS-146	36.3	±0.001	0.72	±0.001	0.261	±0.002	45.6	±0.1	0.8	±0.002	11.6	±0.01	nd	0	0.128	±0.0006	95.4
BS-147	34.1	±0.003	0.75	±0.02	0.307	±0.003	44	±0.89	1.3	±0.005	33.4	±0.002	0.07	±0.0001	0.182	±0.002	114
BS-148	40.4	±0.001	0.13	±0.003	0.283	±0.001	61.1	±1.01	0.5	±0.01	11	±0.001	0.04	±0.0003	0.176	±0.001	114
BS-149	54.1	±0.002	0.35	±0.020	0.366	±0.048	48.4	±0.01	0.7	±0.013	9.71	±0.004	nd	0	0.169	±0.0006	114
BS-150	48.6	±0.020	0.13	±0.001	0.382	±0.007	60.1	±0.029	1	±0.002	8.3	±0.01	0.04	±0.001	0.181	±0.001	119

nd = Not detectable

#### Variation of bioactive phenolics in *Bergenia* species

Comparative quantitative analysis of four *Bergenia* species from different geographical regions of Himalaya showed that the content of investigated phenolics varied significantly among species. The quantity of bergenin with the content range of 28.50–243.00 mg/g was comparatively higher in all 24 samples of *B*. *ligulata* than other species and detected highest (243.00 mg/g) in BS-114 collected from Rajouri (Jammu Kashmir). Similarly, arbutin with the content range of 6.77–77.70 mg/g was comparatively higher in all 16 samples of *B*. *stracheyi* than other species and detected highest (77.00 mg/g) in BS-135 collected from Kullu (Himachal Pradesh). The highest quantity of gallic acid (23.5 mg/g) and ferulic acid (0.53 mg/g) was found in *B*. *ligulata*, whereas protocatechuic acid (0.56 mg/g), chlorogenic acid (23.6 mg/g), catechin (38.30 mg/g) and syringic acid (0.80 mg/g) were found highest in *B*. *ciliata*. The total content of eight bioactive phenolics was detected highest (244.61 mg/g) in *B*. *ligulata* (BS-114) and lowest (8.34 mg/g) in *B*. *ciliata* (BS-31).

The comprehensive quantitative analysis indicated significant intra and interspecies quantitative variation of major bioactive phenolics among four *Bergenia* species. Phenolic compounds reported from medicinal plants possess a wide range of bioactivities. Bergenin (Trihydroxy benzoic acid glycoside) is found as a principle constituent of *B*. *ligulata* and *B*. *ciliata*. It has been reported to have antioxidant, anti-inflammatory, antiviral, antihyperglycemic, immunostimulant, and antipyretic potential [40]. Catechin, which is also present in a significant amount in *Bergenia*, is a flavanol found in a variety of foods and drinks such as fruits, chocolates, wine and tea. It has mainly antioxidant properties [41]. Phenolic acids like gallic acid, protocatechuic acid, chlorogenic acid, and ferulic acid are known to inhibit tumor cells and induce apoptosis [42]. Chlorogenic acid, present in the highest quantity in *B*. *cilliata*, is a is reported to have natural antioxidant as it prevents the dinitrogen trioxide formation by scavenging nitrogen dioxide generated in the human oral cavity. According to an *in-vitro* experiment chlorogenic acid inhibits the mutagenic and carcinogenic N-nitroso compounds and DNA formation in single strand breaks [43]. Thus the present estimates of major bioactive phenolics demonstrated that *B*. *ligulata*, which is one of the key ingredients in various Ayurvedic and Unani herbal formulations, is the best among all species having the highest total content of eight bioactive phenolics (244.61 mg/g) and the highest quantity of bergenin.

#### Variation of bioactive phenolics in *B*. *ciliata*

Out of total 150 samples considered in the present analysis, *B*. *ciliata* had the largest sample size (103). Therefore, a comparative quantitative analysis was carried out for all the samples of *B*. *ciliata*. Bergenin was detected as a major phenolics in as many as 88 samples, which varied from 2.39–72.10 mg/g, except in 15 samples collected from Uttarakhand (UK) (BS-5, BS-6, BS-7, BS-66, BS-67, BS-69, BS-71 and BS74), Himachal Pradesh (HP) (BS-40, BS-41, BS-42 and BS-59) and West Bengal (WB) (BS-80, BS-82 and BS-92). Chlorogenic acid and catechin were found in maximum amount in these 15 samples. Bergenin was found highest (72.10 mg/g) in BS-102 (Penlong, East Sikkim) and lowest (2.39 mg/g) in BS-74 (Uttarkashi, UK). Similarly, the maximum amount of other phenolics i.e. arbutin (20.10 mg/g), gallic acid (6.29 mg/g), protocatechuic acid (0.56 mg/g), chlorogenic acid (23.6 mg/g), catechin (38.30 mg/g), syringic acid (0.80 mg/g) and ferulic acid (0.37 mg/g) was detected in BS-58 (Kullu, HP), BS-100 (Penlong, East Sikkim), BS-86 (Darjeeling, W. Bengal), BS-5 (Nainital, UK), BS-71 (Pithoragarh, UK), BS-72 (Pithoragarh, UK) and BS-102 (Penlong, East Sikkim), respectively. The total concentration of all eight phenolics was found highest (96.15 mg/g) in BS-58 (Kullu, HP) and lowest (8.34 mg/g) in BS-31 (Shimla, HP).

### Comparison of major active phenolics in *Bergenia* species by principal component analysis (PCA)

The principal component analysis was carried out to compare and estimate the quality of phenolics in all the 150 samples of four *Bergenia* species. Triplicate analysis of each sample was used for PCA. Initially, the PCA was done using the quantitative data of eight active phenolics of *B*. *ciliata*, *B*. *ligulata*, *B*. *purpurascens* and *B*. *stracheyi*. PCA score plot (S4 Fig) showed 38.4% variation among all the samples by the first two PCs. As shown in PCA biplot, BS-5 (*B*. *ciliata*), BS-131 (*B*. *purpurascens)*, BS-127 (*B*. *ligulata)* and BS-108 (*B*. *ligulata*) are characteristically different from rest the of samples. The majority of samples showed close similarity in the multidimensional space. Hence, they were together around (0, 0) scores. Many of the samples were placed in the second, third and fourth quadrant of the biplot indicating uncommon features of *Bergenia* species. Since the samples covered wide locations in the Indian Himalayas with different geographical coordinates, it was difficult to classify them effectively based on PCA of the whole sample matrix. Therefore, the PCA was done separately for each of the four species.

**Fig 3:**
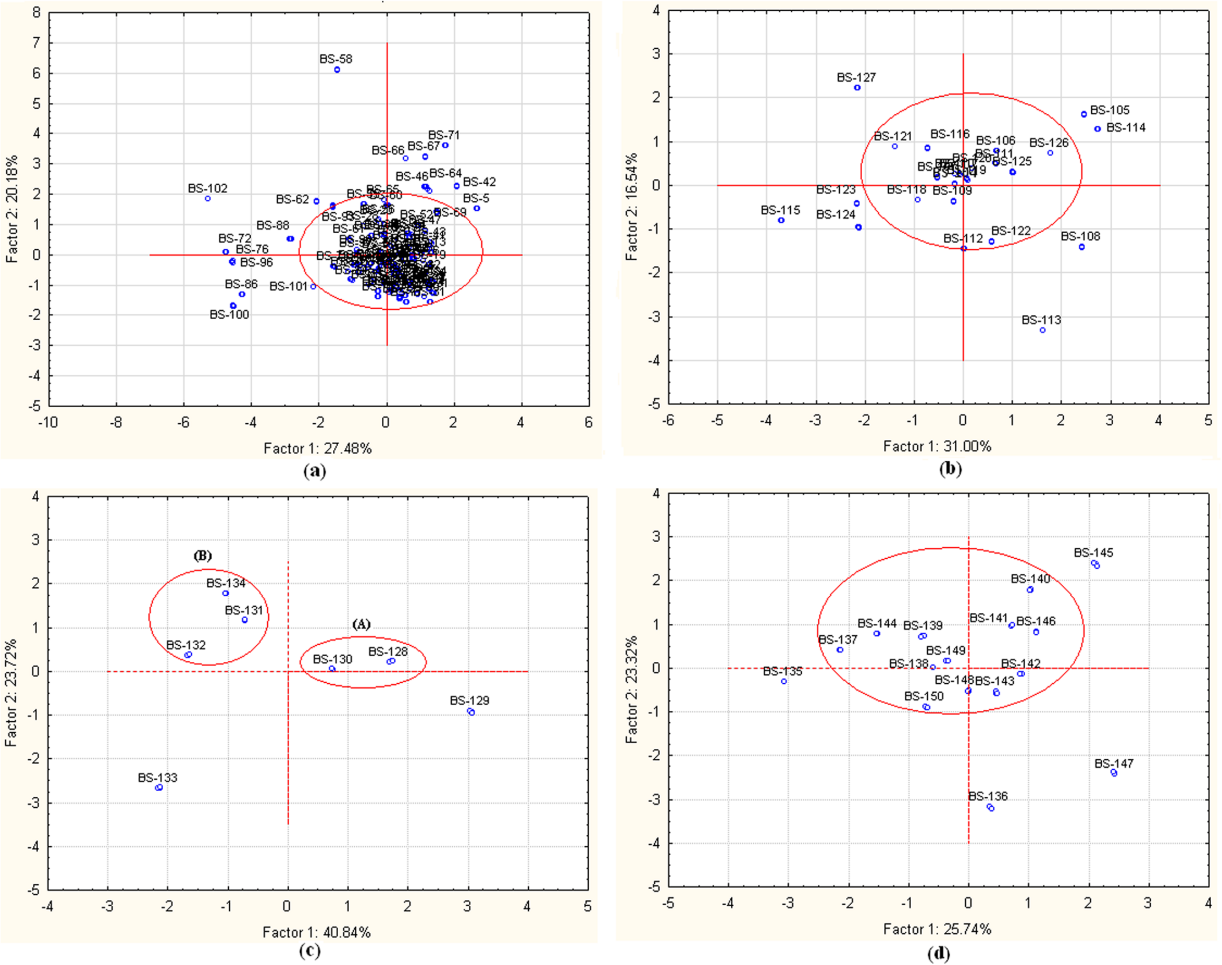
**(A-D).** Principal component analysis score plot for **(A)** 103 samples of *B*. *ciliata*, **(B)** 24 samples of *B*. *ligulata*, **(C)** 7 samples of *B*. *purpurascens* and **(D)** 16 samples of *B*. *stracheyi*. The sample codes are the same as given in **S1 Table**.

The PCA score plot of 103 samples of *B*. *ciliata* showed 47.66% variation by the PC1 and PC2 **(Fig 3A)**. The chemical pattern of the majority of samples was similar, however, 17 samples of *B*. *ciliata* (BS-5, BS-42, BS-46, BS-58, BS-62, BS-64, BS-66, BS-67, BS-71, BS-72, BS-76, BS-86, BS-88, BS-96, BS-100, BS-101 and BS-102) showed different patterns in PCA analysis. The chemical pattern of BS-58 was entirely different from rest of the samples as all the three markers *viz*., catechin (32.4 mg/g), bergenin (39.2 mg/g) and arbutin (20.1 mg/g) were dominant. The characteristic pattern of BS-42, BS-67 and BS-71 was due to the comparatively higher quantity of catechin than the bergenin, which is otherwise the major marker amongst a large number of samples.

The first two PCs explained 47.5% variation in the 24 samples of *B*. *ligulata* (**Fig 3B**). The characteristic variation was observed in 8 samples *viz*., BS-105, BS-108, BS-113, BS-114, BS-115, BS-123, BS-124 and BS-127, whereas rest of the 16 samples showed a similar pattern. The two samples BS-105 (225 mg/g) and BS-114 (243 mg/g) showed close similarity due to the comparatively high amount of bergenin than other samples. BS-123 and BS-124 were distinguished due to catechin content which was 7.7 mg/g and 24.4 mg/g, respectively. BS-127 was unique from the rest of the samples because of a high quantity of gallic acid (23.5 mg/g) which was less than 4 mg/g in other samples.

The two PCs (PC1 and PC2) were able to explain 64.6% variation in the seven samples of *B*. *purpurascens*. All samples of *B*. *purpurascens* showed the dominance of bergenin (41.6–64.8 mg/g) followed by catechin (11.3–33.1 mg/g) and arbutin (5.3–11.1 mg/g). The similarity of the quantitative pattern was found in two subsets: **(A)** BS-128, BS-130 and **(B)** BS-131, BS-132 and BS-134 **(Fig 3C)**.

The PC1 and PC2 explained 49.06% variation in 16 samples of *B*. *stracheyi*. The PC1 score has dominance of bergenin, arbutin and ferulic acid while PC2 score has dominance of syringic acid, chlorogenic acid and arbutin. The factor loading of syringic acid was high, or absent or in negligible quantity in all samples of *B*. *stracheyi*. The BS-135, BS-136, BS-145 and BS-147 have characteristic features. The BS-145 and BS-147 showed distinct features because of the high quantity of catechin in BS-147 and almost 10% of this in BS-145. The BS-135 and BS-136 have characteristic features due to higher quantity of bergenin and arbutin in BS-135 as compared to BS-136, and the highest quantity of chlorogenic acid (4.1 mg/g) in BS-136 than other samples **(Fig 3D)**.

## Conclusions

The newly standardized method we developed and applied in this study has not only facilitated rapid and accurate determination of target analytes but also fulfilled all the criteria of a validated method as per ICH guidelines in terms of sensitivity, precision and accuracy. Results indicated that *B*. *ligulata* collected from Jammu and Kashmir contained highest total content of eight phenolics and the maximum amount of major active marker (Bergenin) which might be medicinally potent regarding cytotoxic and antioxidant activity, whereas *B*. *stracheyi* collected from Kullu (HP) contained the highest quantity of arbutin. However, direct activity relation of phenolics to the geographic location in a large scale sample is a subject of future study. The present study could be helpful in identifying potential accession(s) as a raw material of *Bergenia* species for pharmaceutical industries for preparation of herbal formulations and sustainable utilization of natural resources. The established method also provided a sensitive, accurate and convenient approach for rapid quality control and the establishment of authenticity of *Bergenia* species.

## Supporting information

S1 Table Details of 150 samples of four *Bergenia* species collected from different geographical regions of India. (DOCX)

S1 Fig The MS/MS spectra and the fragmentation scheme of analytes. (A) Arbutin. (B) Gallic acid. (C) Protocatechuic acid.
(TIFF)

S2 Fig The MS/MS spectra and the fragmentation scheme of analytes. (A) Bergenin (B) Chlorogenic acid (C) Catechin.
(TIFF)

S3 Fig The MS/MS spectra and the fragmentation scheme of analytes. (A) Syringic acid (B) Vitexin (C) Ferulic acid.
(TIFF)

S4 Fig Principal component analysis score plot for 150 samples of four *Bergenia* species, *viz*., *B*. *ciliata*, *B*. *ligulata*, *B*. *purpurascens* and *B*. *stracheyi*. (TIFF)
