# Supplementary material for: Major bioactive phenolics in Bergenia species from the Indian Himalayan region: Method development, validation and quantitative estimation using UHPLC-QqQLIT-MS/MS
Source: PLoS One. 2017 Jul 27;12(7):e0180950. doi: 10.1371/journal.pone.0180950 (PMC5531703; doi:10.1371/journal.pone.0180950)
Supplement: S1 Table — (DOCX) [file pone.0180950.s001.docx]

**S1 Table.** Details of 150 samples of four *Bergenia* species collected from different geographical regions of India.

| **S. No.** | **Sample code** | **Voucher specimen number** | ***Bergenia* species** | **Collection Location** | **Latitude** | **Longitude (E)** | **Altitude (m)** | **Date of Collection** |
| --- | --- | --- | --- | --- | --- | --- | --- | --- |
| 1 | BS-1 | 253421 | *B. ciliata* | Kilbari Road, Nainital, UK | 29°24.237' | 079°27.020' | 2281 | 4/24/2013 |
| 2 | BS-2 | 253425 | *B. ciliata* | Kilbari, Nainital, UK | 29°24.302' | 079°26.693' | 2313 | 4/24/2013 |
| 3 | BS-3 | 253429 | *B. ciliata* | Kilbari, Nainital, UK | 29°25.765' | 079°25.515' | 2044 | 4/24/2013 |
| 4 | BS-4 | 253434 | *B. ciliata* | Ghuggukhan, Nainital, UK | 29°25.741' | 079°25.484' | 2053 | 4/24/2013 |
| 5 | BS-5 | 253439 | *B. ciliata* | Kilbari, Nainital, UK | 29°25.311' | 079°26.534' | 2214 | 4/24/2013 |
| 6 | BS-6 | 253440 | *B. ciliata* | Kilbari, Nainital, UK | 29°25.311' | 079°26.534' | 2214 | 4/24/2013 |
| 7 | BS-7 | 253449 | *B. ciliata* | Majkhali, Almora, UK | 29°39.917' | 079°32.148' | 1661 | 4/25/2013 |
| 8 | BS-8 | 253450 | *B. ciliata* | Majkhali, Almora, UK | 29°38.458' | 079°35.161' | 1714 | 4/25/2013 |
| 9 | BS-9 | 253453 | *B. ciliata* | Majkhali, Almora, UK | 29°39.917' | 079°32.148' | 1661 | 4/25/2013 |
| 10 | BS-10 | 253454 | *B. ciliata* | Majkhali, Almora, UK | 29°39.917' | 079°32.148' | 1661 | 4/25/2013 |
| 11 | BS-11 | 253455 | *B. ciliata* | Majkhali, Almora, UK | 29°39.917' | 079°32.148' | 1661 | 4/25/2013 |
| 12 | BS-12 | 253456 | *B. ciliata* | Majkhali, Almora, UK | 29°39.917' | 079°32.148' | 1661 | 4/25/2013 |
| 13 | BS-13 | 253457 | *B. ciliata* | Majkhali, Almora, UK | 29°39.917' | 079°32.148' | 1661 | 4/25/2013 |
| 14 | BS-14 | 253458 | *B. ciliata* | Majkhali, Almora, UK | 29°39.917' | 079°32.148' | 1661 | 4/25/2013 |
| 15 | BS-15 | 253459 | *B. ciliata* | Kosi Katarmal, Almora, UK | 29°37.904' | 079°36.016' | 1404 | 4/26/2013 |
| 16 | BS-16 | 253460 | *B. ciliata* | Dinapani, Almora, UK | 29°39.465' | 079°40.658' | 1810 | 4/26/2013 |
| 17 | BS-17 | 253461 | *B. ciliata* | Dinapani, Almora, UK | 29°39.474' | 079°40.650' | 1810 | 4/26/2013 |
| 18 | BS-18 | 253462 | *B. ciliata* | Dinapani, Almora, UK | 29°39.470' | 079°40.644' | 1810 | 4/26/2013 |
| 19 | BS-19 | 253463 | *B. ciliata* | Binsar WLS, Almora, UK | 29°40.258' | 079°42.811' | 1848 | 4/26/2013 |
| 20 | BS-20 | 253464 | *B. ciliata* | Binsar WLS, Almora, UK | 29°40.258' | 079°42.811' | 1848 | 4/26/2013 |
| 21 | BS-21 | 253467 | *B. ciliata* | Binsar WLS, Almora, UK | 29°40.337' | 079°42.993' | 1881 | 4/26/2013 |
| 22 | BS-22 | 253468 | *B. ciliata* | Binsar WLS, Almora, UK | 29°40.984' | 079°43.735' | 1942 | 4/26/2013 |
| 23 | BS-23 | 253469 | *B. ciliata* | Binsar WLS, Almora, UK | 29°40.991' | 079°43.765' | 1949 | 4/26/2013 |
| 24 | BS-24 | 253471 | *B. ciliata* | Binsar WLS, Almora, UK | 29°40.999' | 079°43.783' | 1979 | 4/26/2013 |
| 25 | BS-25 | 253472 | *B. ciliata* | Binsar WLS, Almora, UK | 29°40.988' | 079°43.789' | 1966 | 4/26/2013 |
| 26 | BS-26 | 253473 | *B. ciliata* | Binsar WLS, Almora, UK | 29°40.988' | 079°43.789' | 1966 | 4/26/2013 |
| 27 | BS-27 | 253478 | *B. ciliata* | Binsar WLS, Almora, UK | 29°41.701' | 079°45.662' | 2264 | 4/26/2013 |
| 28 | BS-28 | 253481 | *B. ciliata* | Binsar WLS, Almora, UK | 29°41.797' | 079°45.469' | 2288 | 4/26/2013 |
| 29 | BS-29 | 253483 | *B. ciliata* | Binsar WLS, Almora, UK | 29°40.991' | 079°43.765' | 1949 | 4/26/2013 |
| 30 | BS-30 | 253484 | *B. ciliata* | Binsar WLS, Almora, UK | 29°40.988' | 079°43.789' | 1966 | 4/26/2013 |
| 31 | BS-31 | 253495 | *B. ciliata* | Shimla, HP | 31°06.480' | 077°10.115' | 2108 | 5/29/2013 |
| 32 | BS-32 | 253496 | *B. ciliata* | Shimla, HP | 31°06.486' | 077°10.109' | 2108 | 5/29/2013 |
| 33 | BS-33 | 253497 | *B. ciliata* | Shimla, HP | 31°06.486' | 077°10.103' | 2107 | 5/29/2013 |
| 34 | BS-34 | 253498 | *B. ciliata* | Shimla, HP | 31°06.480' | 077°10.105' | 2108 | 5/29/2013 |
| 35 | BS-35 | 253499 | *B. ciliata* | Shimla, HP | 31°06.478' | 077°10.102' | 2108 | 5/29/2013 |
| 36 | BS-36 | 253500 | *B. ciliata* | Shimla, HP | 31°06.476' | 077°10.105' | 2106 | 5/29/2013 |
| 37 | BS-37 | 253501 | *B. ciliata* | Shimla, HP | 31°06.447' | 077°10.950' | 2171 | 5/29/2013 |
| 38 | BS-38 | 253502 | *B. ciliata* | Shimla, HP | 31°06.449' | 077°10.953' | 2172 | 5/29/2013 |
| 39 | BS-39 | 253503 | *B. ciliata* | Shimla, HP | 31°06.448' | 077°10.958' | 2171 | 5/29/2013 |
| 40 | BS-40 | 253504 | *B. ciliata* | Kufri, Shimla, HP | 31°06.652' | 077°14.954' | 2506 | 5/29/2013 |
| 41 | BS-41 | 253505 | *B. ciliata* | Kufri, Shimla, HP | 31°06.654' | 077°14.959' | 2506 | 5/29/2013 |
| 42 | BS-42 | 253506 | *B. ciliata* | Kufri, Shimla, HP | 31°06.653' | 077°14.966' | 2506 | 5/29/2013 |
| 43 | BS-43 | 253507 | *B. ciliata* | Kufri, Shimla, HP | 31°06.675' | 077°14.945' | 2506 | 5/29/2013 |
| 44 | BS-44 | 253508 | *B. ciliata* | Kufri to Fagu, Shimla, HP | 31°05.826' | 077°16.380' | 2551 | 5/29/2013 |
| 45 | BS-45 | 253509 | *B. ciliata* | Kufri to Fagu, Shimla, HP | 31°05.825' | 077°16.388' | 2551 | 5/29/2013 |
| 46 | BS-46 | 253510 | *B. ciliata* | Kufri to Fagu, Shimla, HP | 31°05.822' | 077°16.366' | 2551 | 5/29/2013 |
| 47 | BS-47 | 253511 | *B. ciliata* | Kufri to Fagu, Shimla, HP | 31°05.825' | 077°16.359' | 2551 | 5/29/2013 |
| 48 | BS-48 | 253512 | *B. ciliata* | Kamand, Kullu, HP | 31°28.873' | 077°25.429' | 1863 | 5/30/2013 |
| 49 | BS-49 | 253513 | *B. ciliata* | Kamand, Kullu, HP | 31°28.853' | 077°25.431' | 1863 | 5/30/2013 |
| 50 | BS-50 | 253514 | *B. ciliata* | Kamand, Kullu, HP | 31°28.735' | 077°25.505' | 1857 | 5/30/2013 |
| 51 | BS-51 | 253515 | *B. ciliata* | Kamand, Kullu, HP | 31°28.724' | 077°25.506' | 1857 | 5/30/2013 |
| 52 | BS-52 | 253516 | *B. ciliata* | Kullu, HP | 31°28.966' | 077°25.684' | 2088 | 5/30/2013 |
| 53 | BS-53 | 253517 | *B. ciliata* | Kullu, HP | 31°28.966' | 077°25.684' | 2088 | 5/30/2013 |
| 54 | BS-54 | 253518 | *B. ciliata* | Kullu, HP | 31°30.554' | 077°25.198' | 2311 | 5/30/2013 |
| 55 | BS-55 | 253519 | *B. ciliata* | Kullu, HP | 31°30.558' | 077°25.199' | 2311 | 5/30/2013 |
| 56 | BS-56 | 253520 | *B. ciliata* | Kullu, HP | 31°30.549' | 077°25.195' | 2311 | 5/30/2013 |
| 57 | BS-57 | 253521 | *B. ciliata* | Kullu, HP | 31°30.588' | 077°25.205' | 2311 | 5/30/2013 |
| 58 | BS-58 | 253543 | *B. ciliata* | Kothi, Kullu, HP | 32°19.786' | 077°11.832' | 2676 | 5/31/2013 |
| 59 | BS-59 | 253544 | *B. ciliata* | Kothi, Kullu, HP | 32°19.788' | 077°11.825' | 2673 | 5/31/2013 |
| 60 | BS-60 | 253547 | *B. ciliata* | Kothi, Kullu, HP | 32°19.255' | 077°11.645' | 2525 | 5/31/2013 |
| 61 | BS-61 | 253548 | *B. ciliata* | Kothi, Kullu, HP | 32°19.257' | 077°11.648' | 2527 | 5/31/2013 |
| 62 | BS-62 | 253549 | *B. ciliata* | Kothi, Kullu, HP | 32°19.251' | 077°11.659' | 2531 | 5/31/2013 |
| 63 | BS-63 | 253580 | *B. ciliata* | Dewalthal, Pithoragarh, UK | 29°41.198' | 080°13.021' | 1280 | 11-06-2013 |
| 64 | BS-64 | 253581 | *B. ciliata* | Dewalthal, Pithoragarh, UK | 29°41.198' | 080°13.021' | 1280 | 11-06-2013 |
| 65 | BS-65 | 253582 | *B. ciliata* | Dewalthal, Pithoragarh, UK | 29°41.198' | 080°13.021' | 1280 | 11-06-2013 |
| 66 | BS-66 | 253583 | *B. ciliata* | Dewalthal, Pithoragarh, UK | 29°41.198' | 080°13.021' | 1280 | 11-06-2013 |
| 67 | BS-67 | 253584 | *B. ciliata* | Dewalthal, Pithoragarh, UK | 29°41.198' | 080°13.021' | 1280 | 11-06-2013 |
| 68 | BS-68 | 253584 | *B. ciliata* | Dewalthal, Pithoragarh, UK | 29°41.198' | 080°13.021' | 1280 | 11-06-2013 |
| 69 | BS-69 | 253586 | *B. ciliata* | Dewalthal, Pithoragarh, UK | 29°41.198' | 080°13.021' | 1280 | 11-06-2013 |
| 70 | BS-70 | 253587 | *B. ciliata* | Dewalthal, Pithoragarh, UK | 29°41.198' | 080°13.021' | 1280 | 11-06-2013 |
| 71 | BS-71 | 253588 | *B. ciliata* | Dewalthal, Pithoragarh, UK | 29°41.198' | 080°13.021' | 1280 | 11-06-2013 |
| 72 | BS-72 | 256330 | *B. ciliata* | GWLS, Uttarkashi, UK | 31°04.968' | 078°15.334' | 1956 | 04-05-2013 |
| 73 | BS-73 | 256331 | *B. ciliata* | GWLS, Uttarkashi, UK | 31°04.958' | 078°15.331' | 1958 | 04-05-2013 |
| 74 | BS-74 | 256332 | *B. ciliata* | GWLS, Uttarkashi, UK | 31°05.133' | 078°16.046' | 2000 | 04-05-2013 |
| 75 | BS-75 | 256333 | *B. ciliata* | GWLS, Uttarkashi, UK | 31°04.458' | 078°14.744' | 2100 | 04-05-2013 |
| 76 | BS-76 | 256347 | *B. ciliata* | GWLS, Uttarkashi, UK | 31°05.263' | 078°16.196' | 2133 | 04-04-2013 |
| 77 | BS-77 | 256360 | *B. ciliata* | GWLS, Uttarkashi, UK | 31°04.846' | 078°10.265' | 1739 | 04-06-2013 |
| 78 | BS-78 | 256376 | *B. ciliata* | GWLS, Uttarkashi, UK | 31°06.36.5' | 078°14.27.4' | 2220 | 04-06-2013 |
| 79 | BS-79 | 256377 | *B. ciliata* | GWLS, Uttarkashi, UK | 31°06.36.5' | 078°14.27.4' | 2220 | 04-06-2013 |
| 80 | BS-80 | 255715 | *B. ciliata* | Darjeeling, West Bengal | 27°01.771' | 088°01.429' | 2075 | 4/17/2014 |
| 81 | BS-81 | 255716 | *B. ciliata* | Darjeeling, West Bengal | 27°01.771' | 088°01.429' | 2075 | 4/17/2014 |
| 82 | BS-82 | 255717 | *B. ciliata* | Darjeeling, West Bengal | 27°01.771' | 088°01.429' | 2075 | 4/17/2014 |
| 83 | BS-83 | 255718 | *B. ciliata* | Darjeeling, West Bengal | 27°01.771' | 088°01.429' | 2075 | 4/17/2014 |
| 84 | BS-84 | 255719 | *B. ciliata* | Darjeeling, West Bengal | 27°01.771' | 088°01.429' | 2075 | 4/17/2014 |
| 85 | BS-85 | 255720 | *B. ciliata* | Darjeeling, West Bengal | 27°01.771' | 088°01.429' | 2075 | 4/17/2014 |
| 86 | BS-86 | 255721 | *B. ciliata* | Darjeeling, West Bengal | 27°03.23.1' | 088°15.161' | 2087 | 4/17/2014 |
| 87 | BS-87 | 255722 | *B. ciliata* | Darjeeling, West Bengal | 27°03.23.1' | 088°15.161' | 2087 | 4/17/2014 |
| 88 | BS-88 | 255723 | *B. ciliata* | Darjeeling, West Bengal | 27°03.23.1' | 088°15.161' | 2087 | 4/17/2014 |
| 89 | BS-89 | 255724 | *B. ciliata* | Darjeeling, West Bengal | 27°03.23.1' | 088°15.161' | 2087 | 4/17/2014 |
| 90 | BS-90 | 255725 | *B. ciliata* | Darjeeling, West Bengal | 27°03.23.1' | 088°15.161' | 2087 | 4/17/2014 |
| 91 | BS-91 | 255726 | *B. ciliata* | Darjeeling, West Bengal | 27°03.23.1' | 088°15.161' | 2087 | 4/17/2014 |
| 92 | BS-92 | 255727 | *B. ciliata* | Darjeeling, West Bengal | 27°03.23.1' | 088°15.161' | 2087 | 4/17/2014 |
| 93 | BS-93 | 255752 | *B. ciliata* | Gangtok, East Sikkim, Sikkim | 27°20.445' | 088°36.726' | 1650 | 4/25/2014 |
| 94 | BS-94 | 255751 | *B. ciliata* | Gangtok, East Sikkim, Sikkim | 27°20.441' | 088°36.727' | 1648 | 4/25/2014 |
| 95 | BS-95 | 255801 | *B. ciliata* | Penlong, East Sikkim, Sikkim | 27°22.482' | 088°37.352' | 1645 | 4/28/2014 |
| 96 | BS-96 | 255802 | *B. ciliata* | Penlong, East Sikkim, Sikkim | 27°22.459' | 088°37.327' | 1645 | 4/28/2014 |
| 97 | BS-97 | 255803 | *B. ciliata* | Penlong, East Sikkim, Sikkim | 27°22.452' | 088°37.323' | 1645 | 4/28/2014 |
| 98 | BS-98 | 255804 | *B. ciliata* | Penlong, East Sikkim, Sikkim | 27°22.445' | 088°37.328' | 1645 | 4/28/2014 |
| 99 | BS-99 | 255805 | *B. ciliata* | Penlong, East Sikkim, Sikkim | 27°22.443' | 088°37.315' | 1645 | 4/28/2014 |
| 100 | BS-100 | 255806 | *B. ciliata* | Penlong, East Sikkim, Sikkim | 27°22.446' | 088°37.305' | 1645 | 4/28/2014 |
| 101 | BS-101 | 255807 | *B. ciliata* | Penlong, East Sikkim, Sikkim | 27°22.440' | 088°37.300' | 1645 | 4/28/2014 |
| 102 | BS-102 | 255808 | *B. ciliata* | Penlong, East Sikkim, Sikkim | 27°22.435' | 088°37.312' | 1645 | 4/28/2014 |
| 103 | BS-103 | 255809 | *B. ciliata* | Penlong, East Sikkim, Sikkim | 27°22.427' | 088°37.271' | 1645 | 4/28/2014 |
| 104 | BS-104 | 255843 | *B. ligulata* | Rajouri, J & K | 33°35.059' | 074°21.483' | 2025 | 8/22/2014 |
| 105 | BS-105 | 255844 | *B. ligulata* | Rajouri, J & K | 33°35.059' | 074°21.483' | 2025 | 8/22/2014 |
| 106 | BS-106 | 255845 | *B. ligulata* | Rajouri, J&K | 33°35.655' | 074°21.854' | 1769 | 8/22/2014 |
| 107 | BS-107 | 255846 | *B. ligulata* | Rajouri, J&K | 33°35.655' | 074°21.854' | 1769 | 8/22/2014 |
| 108 | BS-108 | 255847 | *B. ligulata* | Rajouri, J&K | 33°35.655' | 074°21.854' | 1769 | 8/22/2014 |
| 109 | BS-109 | 255848 | *B. ligulata* | Rajouri, J&K | 33°35.655' | 074°21.854' | 1769 | 8/22/2014 |
| 110 | BS-110 | 255849 | *B. ligulata* | Rajouri, J&K | 33°35.655' | 074°21.854' | 1769 | 8/22/2014 |
| 111 | BS-111 | 255850 | *B. ligulata* | Rajouri, J&K | 33°35.655' | 074°21.854' | 1769 | 8/22/2014 |
| 112 | BS-112 | 255851 | *B. ligulata* | Rajouri, J&K | 33°35.655' | 074°21.854' | 1769 | 8/22/2014 |
| 113 | BS-113 | 255852 | *B. ligulata* | Rajouri, J&K | 33°35.655' | 074°21.854' | 1769 | 8/22/2014 |
| 114 | BS-114 | 255853 | *B. ligulata* | Rajouri, J&K | 33°35.349' | 074°21.503' | 2144 | 8/22/2014 |
| 115 | BS-115 | 255854 | *B. ligulata* | Rajouri, J&K | 33°35.349' | 074°21.503' | 2144 | 8/22/2014 |
| 116 | BS-116 | 255855 | *B. ligulata* | Rajouri, J&K | 33°35.349' | 074°21.503' | 2144 | 8/22/2014 |
| 117 | BS-117 | 255856 | *B. ligulata* | Rajouri, J&K | 33°35.349' | 074°21.503' | 2144 | 8/22/2014 |
| 118 | BS-118 | 255857 | *B. ligulata* | Rajouri, J&K | 33°35.349' | 074°21.503' | 2144 | 8/22/2014 |
| 119 | BS-119 | 255858 | *B. ligulata* | Rajouri, J&K | 33°35.349' | 074°21.503' | 2144 | 8/22/2014 |
| 120 | BS-120 | 255859 | *B. ligulata* | Rajouri, J&K | 33°35.349' | 074°21.503' | 2144 | 8/22/2014 |
| 121 | BS-121 | 255860 | *B. ligulata* | Rajouri, J&K | 33°35.349' | 074°21.503' | 2144 | 8/22/2014 |
| 122 | BS-122 | 255861 | *B. ligulata* | Rajouri, J&K | 33°35.349' | 074°21.503' | 2144 | 8/22/2014 |
| 123 | BS-123 | 255862 | *B. ligulata* | Rajouri, J&K | 33°35.349' | 074°21.503' | 2144 | 8/22/2014 |
| 124 | BS-124 | 255863 | *B. ligulata* | Rajouri, J&K | 33°35.349' | 074°21.503' | 2144 | 8/22/2014 |
| 125 | BS-125 | 255864 | *B. ligulata* | Rajouri, J&K | 33°35.349' | 074°21.503' | 2144 | 8/22/2014 |
| 126 | BS-126 | 255865 | *B. ligulata* | Rajouri, J&K | 33°35.349' | 074°21.503' | 2144 | 8/22/2014 |
| 127 | BS-127 | 255866 | *B. ligulata* | Rajouri, J&K | 33°35.349' | 074°21.503' | 2144 | 8/22/2014 |
| 128 | BS-128 | 255796 | *B. purpurascens* | North Sikkim, Sikkim | 27°54.460' | 088°29.460' | 4050 | 4/27/2014 |
| 129 | BS-129 | 255797 | *B. purpurascens* | North Sikkim, Sikkim | 27°54.465' | 088°29.462' | 4052 | 4/27/2014 |
| 130 | BS-130 | 255798 | *B. purpurascens* | North Sikkim, Sikkim | 27°54.411' | 088°29.422' | 4048 | 4/27/2014 |
| 131 | BS-131 | 255799 | *B. purpurascens* | North Sikkim, Sikkim | 27°54.415' | 088°29.438' | 4040 | 4/27/2014 |
| 132 | BS-132 | 255800 | *B. purpurascens* | North Sikkim, Sikkim | 27°54.406' | 088°29.423' | 4044 | 4/27/2014 |
| 133 | BS-133 | 255810 | *B. purpurascens* | North Sikkim, Sikkim | 27°54.402' | 088°29.456' | 4041 | 4/27/2014 |
| 134 | BS-134 | 255811 | *B. purpurascens* | North Sikkim, Sikkim | 27°54.422' | 088°29.475' | 4043 | 4/27/2014 |
| 135 | BS-135 | 253525 | *B. stracheyi* | Jalori Pass, Kullu, HP | 31°32.245' | 077°22.475' | 3115 | 5/30/2013 |
| 136 | BS-136 | 253527 | *B. stracheyi* | Jalori Pass, Kullu, HP | 31°32.254' | 077°22.488' | 3137 | 5/30/2013 |
| 137 | BS-137 | 255827 | *B. stracheyi* | Khillenmarg, Baramulla, J&K | 34°02.027' | 074°21.268' | 2211 | 8/19/2014 |
| 138 | BS-138 | 255828 | *B. stracheyi* | Khillenmarg, Baramulla, J&K | 34°02.027' | 074°21.268' | 2211 | 8/19/2014 |
| 139 | BS-139 | 255829 | *B. stracheyi* | Khillenmarg, Baramulla, J&K | 34°02.027' | 074°21.268' | 2211 | 8/19/2014 |
| 140 | BS-140 | 255830 | *B. stracheyi* | Khillenmarg, Baramulla, J&K | 34°02.027' | 074°21.268' | 2211 | 8/19/2014 |
| 141 | BS-141 | 255831 | *B. stracheyi* | Khillenmarg, Baramulla, J&K | 34°02.027' | 074°21.268' | 2211 | 8/19/2014 |
| 142 | BS-142 | 255835 | *B. stracheyi* | Khillenmarg, Baramulla, J&K | 34°02.027' | 074°21.268' | 2211 | 8/19/2014 |
| 143 | BS-143 | 255836 | *B. stracheyi* | Khillenmarg, Baramulla, J&K | 34°02.027' | 074°21.268' | 2211 | 8/19/2014 |
| 144 | BS-144 | 255837 | *B. stracheyi* | Khillenmarg, Baramulla, J&K | 34°02.027' | 074°21.268' | 2211 | 8/19/2014 |
| 145 | BS-145 | 255838 | *B. stracheyi* | Khillenmarg, Baramulla, J&K | 34°02.027' | 074°21.268' | 2211 | 8/19/2014 |
| 146 | BS-146 | 255839 | *B. stracheyi* | Khillenmarg, Baramulla, J&K | 34°02.027' | 074°21.268' | 2211 | 8/19/2014 |
| 147 | BS-147 | 255840 | *B. stracheyi* | Khillenmarg, Baramulla, J&K | 34°02.027' | 074°21.268' | 2211 | 8/19/2014 |
| 148 | BS-148 | 255841 | *B. stracheyi* | Khillenmarg, Baramulla, J&K | 34°02.027' | 074°21.268' | 2211 | 8/19/2014 |
| 149 | BS-149 | 255842 | *B. stracheyi* | Khillenmarg, Baramulla, J&K | 34°02.027' | 074°21.268' | 2211 | 8/19/2014 |
| 150 | BS-150 | 253599 | *B. stracheyi* | Khillenmarh, Baramulla, J&K | 34°02.482' | 074°20.803' | 2224 | 8/19/2014 |

BWLS; Binsar wildlife sanctuary, GWLS**;** Govind wildlife sanctuary, UK; Uttarakhand, HP; Himachal Pradesh, J&K; Jammu and Kashmir
